# Detection of *Helicobacter pylori glmM* gene in bovine milk using Nested polymerase chain reaction

**DOI:** 10.14202/vetworld.2015.913-917

**Published:** 2015-07-26

**Authors:** Eyman Y. Osman, A. M. S. El-Eragi, Abuobeida M. Musa, Salma B. El-Magboul, Magdi B. A/Rahman, Abdelmounem E. Abdo

**Affiliations:** 1 Department of Pathology and Diagnosis, Veterinary Research Institute, P. O. Box 8067, ALAmart (St. 1), Khartoum, Sudan; 2 Department of Microbiology, College of Medicine, Bisha University, P.O. Box: 1140, KSA; 3 Department of Health, Maliha Municipality, P. O. Box: 13888, Sharjah, UAE; 4 Department of Molecular Biology, Veterinary Research Institute, P. O. Box 8067, ALAmart (St. 1), Khartoum, Sudan; 5 Department of Pathology and Diagnosis, Veterinary Research Institute. P. O. Box 8067, ALAmart (St. 1), Khartoum, Sudan; 6 Department of Gastroenterology, Director of the National Centre for Gastrointestinal and liver disease, Ibnsina Hospital, Alamarat (St. 17) postal code 122117 P. O. Box: 15004, Khartoum, Sudan

**Keywords:** bovine, *Helicobacter pylori*, Khartoum, milk, Nested polymerase chain reaction, *glmM* gene

## Abstract

**Aim:**

The aim was to detect the *glmM* gene of *Helicobacter pylori* (*H. pylori*) in cow’s milk from different dairy farms in Khartoum State using Nested polymerase chain reaction (PCR).

**Materials and Methods:**

A total of 50 milk samples were collected from different dairy farms in Khartoum State (13 from Khartoum, 24 Khartoum North, and 13 from Omdurman Provinces).

**Results:**

The generated results showed that 11/50 (22%) were harboring the investigated *H. pylori glmM* gene in Khartoum State (1/13 [7.7%] Khartoum, 9/24 [37.5%] Khartoum North, and 1/13 [7.7%] Omdurman provinces, respectively).

**Conclusion:**

To the best of our knowledge, this was the first report on the detection of *H. pylori glmM* gene in cattle milk in Khartoum State. Nonetheless, the high percentages of *H. pylori* DNA detection in milk opened new avenues toward exploring the risk of human infection with *H. pylori* through the consumption of raw milk.

## Introduction

*Helicobacter pylori* (*H. pylori*) infection constitutes a public health concern in developed and developing countries [1,2], since it was associated with chronic gastritis, gastric and duodenal ulcers, and gastric adenocarcinoma in humans, as well as mucosa-associated lymphoid tissue lymphoma [3-6]. In the year 1994, the International Agency for Research on Cancer of WHO classified *H. pylori* as a Type I, or definite carcinogen to humans and reconfirmed this classification in 2009 [7].

Cow’s milk is usually consumed as human food, especially by children. Therefore, one of the suggested theories is the transmission of *H. pylori* through milk from animals to human beings. Some epidemiologic studies have reported the presence and survival of *H. pylori* in raw and pasteurized milk and milk products of cow, sheep, and goat directly or artificially inoculated was investigated either using Nested or semi-Nested PCR or by culturing methods or by the two techniques together [8-12]. Furthermore, epidemiologic data have shown higher prevalence in shepherds and their families than in the general population [13,14].

Nested PCR was already employed for the detection of *H. pylori* *glmM* gene from water and was reported to be as a highly sensitive and specific method for the rapid detection and screening of sheep, goat and cow milk [11-15].

The *ureC* gene of *H. pylori* encodes for the phosphoglucosamine mutase catalyzing the interconversion of GlcN-6-phosphate (GlcN-6-P) and GlcN-1-P isomers required for the biosynthesis of lipopolysaccharides and peptidoglycan. This gene has been shown to play an essential and unique role for *H. pylori* growth and survival [16-19]. It is presently named as *glmM* rather than *ureC* since it’s unrelated to urease production [17]. The aim of this study was to detect the *glmM* gene of *H. pylori* in cow’s milk from different dairy farms in Khartoum State using Nested PCR.

## Materials and Methods

### Ethical approval

The milk samples were collected aseptically with adequate precautionary measures to minimize pain and/or discomfort to the animals and carried out in accordance with the Sudan animal welfare laws. Furthermore, the human biopsies were taken under high precautionary measures and include approval of the ethical committee and informed consent was obtained from all of study patients to use these biopsies in this research.

### Study area

Khartoum State is the capital of Sudan and includes “Khartoum, Khartoum North, and Omdurman Localities,” comprising the semi-arid zone between the latitude 15.08°N to 16.39°N and longitude 31.36°E to 34.25°E. This study was carried out in the Department of Molecular Biology, Veterinary Research Institute, Khartoum, Sudan.

### Specimen collection

A total of 50 fresh raw cow’s milk samples were collected from different dairy farms in Khartoum State, Sudan. The samples were put into sterile ice containers and promptly delivered to the laboratory. Furthermore, four biopsies were taken from four human patients (two biopsies each) who were undergoing upper gastroduodenal endoscopy and tested by rapid urease test (RUT). After test, the human’s biopsies were checked for the presence of *glmM* gene using Nested PCR, and used later as a positive control for the collected milk samples.

### Bacterial culture

A total of 50 fresh milk samples were cultured immediately after collection into broth medium firstly and then transferred to solid media. Collected milk samples were cultivated bacteriologically for the isolation of *H. pylori* using the following protocol: 3 ml of each sample were added to 10 ml of Brain Heart Infusion Broth supplemented with 5% of horse serum and Campylobacter Selective Supplement, Skirrow powder (Cat. No. CSS80004, Biolab, Hungary) and amphotericin B solution (250 µg/mL, 1 ml/L) (Cat. No. A2942, Sigma). This supplement contain polymyxin B (0,2 mg/vial), Trimethoprim (2.5 mg/vial), and vancomycin (5 mg/vial), reconstituted with 4 ml of sterile distilled water and added aseptically to 500 ml sterile broth at 50°C, mixed well before pouring and incubated at 37°C under microaerophilic conditions. After 3-5 days incubation in 0.1 ml of the selective enrichment broth, the culture was plated on brain heart infusion agar, blood agar base (No. 2) and egg yolk emulsion agar supplemented with 5% defibrinated sheep blood, campylobacter selective supplement, skirrow powder (Cat. No. CSS80004, Biolab, Hungary), and amphotericin B solution (250 µg/mL, 1 ml/L) (Cat. No. A2942, Sigma) were added aseptically to 500 ml sterile broth at 50°C, mixed well before pouring and incubated for 3-5 days at 37°C under microaerophilic conditions according to Poms and Tatini [20].

### DNA extraction

Using 1 ml from each milk sample (50 samples), DNA was extracted using QIAamp Blood Mini Kit (Cat. No. 51104, QIAGEN), with slightly modifying the supplier’s instructions according to Quaglia *et al*. [11].

### Oligonucleotide primers

Oligonucleotide primers (Cat. No. 011101853 Tib Molbiol Syntaborhesel GmbH, Berlin, Germany) as published by Quaglia *et al*. [11] were used throughout this investigation. Outer oligonucleotide primers Hp 1 and Hp 2 were used to amplify *H. pylori* *glmM* gene at 294 bp region:

**Hp 1:** 5’-AAGCTTTTAGGGGTGTTAGGGGTTT-3′

**Hp 2:** 5’-AAGCTTACTTTCTAACACTAAACGC-3′

Internal primers Hp 3 and Hp 4 were used to amplify a 252 bp region located 21 base pairs:

**Hp 3:** 5’-CTTTCTTCTCAAGCGGTTGTC-3′

**Hp 4:** 5’-CAAGCCATCGCCGGTTTTAGC-3′

### Nested PCR protocol

A volume of 5 µl of each extracted DNA were amplified in 50 µl reaction mixture containing ×10 Taq buffer BD (0.8 M Tris-HCl, 0.2 M (NH_4_)_2_ SO_4_), 25 mM MgCl_2_), 200 µM dNTPs mix, 0.5 µM of each primer, and 5 U/µl FIREPol^®^ Taq DNA polymerase (Cat. No. 01-01-00500, Solis BioDyne, Riia).

PCR amplification was performed according to the Quaglia *et al*. [11] protocol as follows: 95°C for 3 min and 35 cycles at 93°C for 1 min, 58°C for 1 min, 72°C for 2 min followed by 72°C for 7 min. After the amplification, 2 µl of the final product were transferred to the second PCR run. The reaction mixture containing the PCR product of the first run was re-amplified for 30 cycles using primers Hp 3 and Hp 4 under the following conditions: 95°C for 2 min and 30 cycles at 94°C for 1 min, 60°C for 2 min, 72°C for 3 min followed by 72°C for 7 min.

Nested-PCR products were visualized under UV transillumination following electrophoresis on 1.5% agarose gel stained with ethidium bromide 0.5 mg/ml using Gene Ruler VC 100-bp DNA Ladder as a reference standard (Cat. No. NL 1401, Vivantis Company, Malaysia).

### Positive control

The positive control was prepared by extraction of DNA and conducting *glmM* Nested PCR for three positive a RUT human patients’ biopsies (two biopsies each).

## Results

### Nested PCR results

**Figure-1:**
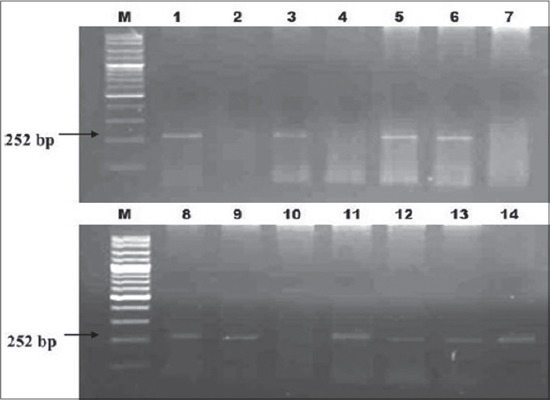
Nested PCR of *glmM* gene of *Helicobacter pylori* in raw Cow milk, Lane M = 100 bp ladder, Lane 1 = positive control, Lane 7 = negative control, Lane 3, 5, 6, 8, 9, 11, 12, 13, and 14 = positive milk samples and lane 2, 4, and 10 = negative milk samples.

**Figure-2:**
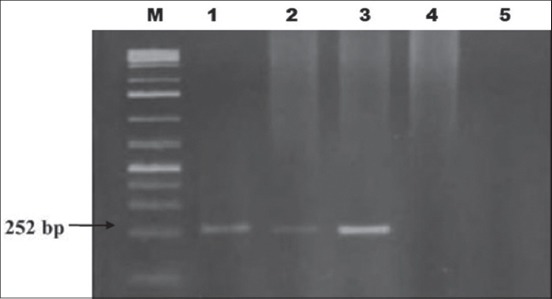
Nested PCR of *glmM* gene of *Helicobacter pylori* in four human gastric biopsy specimens, Lane M = 100 bp, Ladder, Lane 1, 2, and 3 = positive gastric biopsies, Lane 4 = negative gastric biopsies, and Lane 5 = negative control.

The test revealed that 11/50 (22%) raw cow’s milk samples from different dairy farms in Khartoum State were positive. The positive samples were distributed in the State as follows; Khartoum 1/13 (7.7%), Khartoum North 9/24 (37.5%), and 1/13 (7.7%) Omdurman, respectively (Figure-1). Moreover, ¾ of the human gastric biopsies were positive for *H. pylori* while the 4^th^ one was negative. These positive biopsies were used as positive controls in Nested PCR for the detection of *H. pylori* *glmM* (*ureC*) gene. The results of the gastric biopsies on Nested PCR were identical to those of the RUT (Figure-2).

## Discussion

This study was formulated for the detection of *H. pylori glmM* (*ureC*) gene by nested PCR. The prevalence rate was 22% from fresh raw cow’s milk in Khartoum State. However, the highest rate (37.5%) was observed on Khartoum North and an equal rate (7.7%) was detected in Khartoum and Omdurman. Whereas scandinavian researchers found *H. pylori* in 60% of 38 sheep gastric tissue [8]. In the other study which was conducted in Japan, *H. pylori* was found in 72.2% of cow raw milk specimens [9]. The Italian survey on 400 milk samples by nested PCR assay indicated that the prevalence of this bacterium in cow, sheep and goat populations were 50%, 33% and 25.6% respectively [10]. Furthermore, Rahimi and Kheirabadi declared *H. pylori* existed in 12.2% of sheep, 8.7% of goat, and 14.1% of cow milk by PCR method [21]. In addition, Hassan *et al.*, [22] was reported that the prevalence of *H. pylori* in cow’s and sheep’s gastric biopsies was 3% and 16%, respectively, in Iran. Furthermore, Angelidis *et al*. [23] detected *H. pylori* in 20% of bovine bulk tank milk by fluorescence in situ hybridization. On the contrary, Bianchini *et al*. [24], isolated other Helicobacteraceae than *H. pylori* from raw milk of dairy cattle by cultural methods and confirmed results by Nested PCR with prevalence of 1.8% (3/163 samples), while Mousavi *et al.*, reported a prevalence of isolated *H. pylori* of 19.8% (16.66%, 35%, 28%, 15%, and 13.33%, from bovine, ovine, caprine, buffalo, and camel raw milk samples, respectively) and 19.2% of dairy products samples based on cultural method, Nested PCR, and antimicrobial susceptibility testing which revealed high presence of antibiotic-resistant isolated strains of *H. pylori* in milk and dairy products to ampicillin (84.4%), tetracycline (76.6%), erythromycin (70.5%), and metronidazole (70%), [25]. Our finding were in agreement with those of Quaglia, *et al*. [11]., Ghasemian *et al.*, [26] and Rahimi and Kheirabadi, [21] who reported that *glm M* (*ureC*) gene nested PCR is a highly sensitive and specific for detection of *H. pylori* from cows, goats, sheep, camels, and buffalo milk samples. Therefore, all those animals mentioned and their products may act as a reservoir of *H. pylori* and transmitted this bacterium to humans.

The ability of *H. pylori* to survive in an acid pH environment is urea dependent [27,28] and since urea is present in milk [21], the urea-dependent acid resistance of *H. pylori* may account for the long-term survival of *H. pylori* in this foodstuff [29]. This was in line with the finding of Quaglia, *et al.*, [12] who reported that *H. pylori* was survival for 9 days in pasteurized milk and 12 days in ultrahigh temperature milk stored at 4°C. However, the presence of *H. pylori* *glmM* gene in milk in the present study in Khartoum State might be due to the poor hygienic management in dairy farms and ability of this bacterium to survive in milk for a relatively long term. Several investigations showed a number of animals, mostly living in human environment, had *H. pylori* in their stomach and in their production and, therefore, to be involved in the transmission of this bacterium [11,21,26,30]. Despite the fact that the transmission pathway of *H. pylori* is unclear, but the poor hygienic management in dairy farms might contribute to spread of bacterium from animal to animal through udder teats from contaminated milking machines or by milkers’ hands and towels. Furthermore, contaminated manure, bedding, soil, and water are might be transferred organism to the udder during the milking process or shortly thereafter. Also milk could become contaminated during production or because of low hygiene after the open of package [25], so insufficient post-processing hygienic management of the milk, can carry the contamination of the matrix by humans [12]. Therefore, we need food safety and quality standards (good dairy farming practices, good manufacturing practices, and the hazard analysis and critical control point system to be applied and performed in consumed milk by a human.

Concerning the *glmM* (*ureC*) gene Nested PCR, which was reported to be the most sensitive and specific for the detection of *H. pylori* in gastric biopsies by Saurabh *et al.*, [16]. The findings of the present investigation supported this statement when we examined positive RUT gastric biopsies using the above-mentioned technique. In reference to this statement, the RUT positive gastric biopsies were used in this study as positive controls. Furthermore, single round PCR may give positive amplification only when more than 70 bacterial cells are present in a given biopsy sample, while Nested PCR is capable of detecting the bacterium as low as 3 cells only. Therefore, Nested PCR may be proposed as the gold standard for detection of *H. pylori*. [16].

The failure of *H. pylori* recovery from fresh raw milk samples by cultural methods in this study was supported by the findings of Dore *et al.*, [8]; Fujimura *et al.*,[9]; Ghasemian *et al.*,[26]; and Angelidis *et al.*,[23], who reported that, *H. pylori* has rarely been isolated from raw and pasteurized milk samples. In addition, other studies failed to isolate *H. pylori* from raw cow [10], sheep [26], and goat [31] milk samples.

It has been reported that *H. pylori* change to three different morphological forms under environmental stress. These forms include the viable spiral, viable coccoid, and nonviable degenerative forms. While the viable spiral forms are culturable, virulent, and infectious, and induce inflammation in experimental animals. The viable coccoid forms are nonculturable, less virulent, and less likely to colonize, and induce inflammation in experimental animals. On the other hand, the nonviable degenerative forms are dying forms of *H. pylori* [32]. Therefore, the present failure in isolating the bacterium can be attributed to the fact that *H. pylori* can survive for short periods of time in milk and that it may have changed into the other non-cultivable forms during sample transit from field to laboratory. This has been strongly supported by the findings of Quaglia *et al*. [12]. Moreover, the method employed for *H. pylori* isolation may lack sufficient sensitivity to recover very low numbers of *H. pylori* due to the presence of viable nonculturable form (10 CFU per ml of liquid samples to recover at least one colony per plate) [20-23,26-29]. Furthermore, isolation is extremely difficult due to the presence of accompanying microflora and very low numbers of *H. pylori* in foodstuffs [33].

To the best of our knowledge, the present study was the first report that demonstrated *H. pylori* DNA in bovine milk in Khartoum State. Nonetheless, the presence of *H. pylori* DNA of milk analyzed in this work, open new avenues toward exploring the risk of human infection with *H. pylori* through the consumption of raw milk and bovine may play a role as a natural reservoir of the microorganism.

## Conclusion

The present investigation demonstrated the presence of *H. pylori* *glmM* (*ureC*) gene in 11/50 (22%) raw cow’s milk samples from different dairy farms in Khartoum State, Sudan. This study’s outcome provides evidence on the possible transmission of *H. pylori* from milk to human, through consumption of raw milk.

## Authors’ Contributions

AMSE helped in the study design and revised the manuscript. SBM helped EYO to conduct the nested PCR and analyzed the results. AEA collected the human patient’s biopsies using endoscopy and carried out the rapid urease test (RUT). EYO & AMM Collected the bovine milk samples and drafted the manuscript. MBAR helped in the revision of the manuscript. All authors read and approved the final manuscript.

## References

[1] Hunt C.R.H, Xiao S.D, Megraud F, Leon-Barua R, Bazzoli F, van der Merwe S, Vaz Coelho L.G, Fock K.M, Fedail S, Cohen H, Malfertheiner P, Vakil N, Hamid S, Goh K.L, Wong B.C.Y, Krabshuis J.H (2011). Helicobacter pylori in developing countries. World gastroenterology organisation global guideline. J. Gastrointest. Liver.

[2] Leonardo H, Eusebi L.H, Zagari R.M, Bazzoli F (2014). Epidemiology of *Helicobacter pylori* infection. Helicobacter.

[3] Marina de Bernard and Christine Josenhans (2014). Pathogenesis of Helicobacter pylori Infection. Helicobacter.

[4] Bauer B, Meyer T.F (2011). The human gastric pathogen *Helicobacter pylori* and its association with gastric cancer and ulcer disease. Ulcers.

[5] Neumeister P, Troppan K, Raderer M (2015). Management of gastric mucosa-associated lymphoid tissue lymphoma. J. Dig. Dis.

[6] Meine G.C, Rota C, Dietz J, Sekine S, Prolla J.C (2011). Relationship between cagA-positive *Helicobacter pylori* infection and risk of gastric cancer:A case control study in Porto Alegre, RS, Brazil. Arq Gastroenterol.

[7] IARC *Helicobacter pylori* Working Group (2014). *Helicobacter pylori* Eradication as a Strategy for Preventing Gastric Cancer.

[8] Dore M.P, Sepulveda A.R, El-Zimaity H, Yamaoka Y, Osato M.S, Mototsugu K, Nieddu A.M, Realdi G, Graham D.Y (2001). Isolation of *Helicobacter pylori* from sheep—implications for transmission to humans. Am. J. Gastroenterol.

[9] Fujimura S, Kawamura T, Kato S, Tateno H, Watanabe A (2002). Detection of *Helicobacter pylori* in cow’s milk. Lett. Appl. Microbiol.

[10] Quaglia C.N, Dambrosio A, Normanno G, Parisi A, Patrono R, Ranieri G, Rella A, Celano G.V (2008). High occurrence of *Helicobacter pylori* in raw goat, sheep and cow milk inferred by glmM gene:A risk of food-borne infection. Int. J. Food Microbiol.

[11] Quaglia N.C, Dambrosio A, Normanno G, Celano G.V (2009). Evaluation of a nested-PCR assay based on the phosphoglucosamine mutase gene (glmM) for the detection of *Helicobacter pylori* from raw milk. Food Control.

[12] Quaglia N.C, Dambrosio A, Normanno G, Parisi A, Firinu A, Lorusso V (2007). Survival of *Helicobacter pylori* in artificially contaminated ultrahigh temperature and pasteurized milk. Lancet.

[13] Dore M.P, Bilotta M, Vaira D, Manca A, Massarelli G, Leandro G, Atzei A, Pisanu G, Graham D. Y, Realdi G (1999). High prevalence of *Helicobacter pylori* infection in shepherds. Dig. Dis. Sci.

[14] Plonka M, Bielanski W, Konturek S.J, Targosz A, Sliwowski Z, Dobrzanska M, Kaminska A, Sito E, Konturek P.C, Brzozowski T (2006). *Helicobacter pylori* infection and serum gastrin, ghrelin and leptin in children of polish shepherds. Dig. Liver Dis.

[15] Bahrami A.R, Rahimi E, Safaei H.G (2013). Detection of *Helicobacter pylori* in city water, dental units’ water, and bottled mineral water in Isfahan, Iran. Sci. World J.

[16] Patel S.K, Mishra G.N, Pratap C.B, Jain A.K, Nath G (2014). *Helicobacter pylori* is not eradicated after triple therapy:A nested PCR based study. BioMed. Res. Int.

[17] De Reuse H, Labigne A, Mengin-Lecreulx D (1997). The *Helicobacter pylori* ureC gene codes for a phosphoglucosamine mutase. J. Bacteriol.

[18] Labigne A, Cussac V, Courcoux P (1991). Shuttle cloning and nucleotide sequences of *Helicobacter pylori* genes responsible for urease activity. J. Bacteriol.

[19] Mengin-Lecreulx D, van Heijenoort J (1996). Characterization of the essential glmM gene encoding phosphoglucosamine mutase in *Escherichia coli*. J. Biol. Chem.

[20] Poms R.E, Tatini S.R (2001). Survival of *Helicobacter pylori* in ready-to-eat foods at 4°C. Int. J. Food Microbiol.

[21] Rahimi E, Kheirabad E. K (2012). Detection of *Helicobacter pylori in bovine* buffalo, camel, ovine, and caprine milk in Iran. Foodborne Pathog. Dis.

[22] Momtaz H, Dabiri H, Souod N, Gholami M (2014). Study of *Helicobacter pylori* genotype status in cows, sheep, goats and human beings. BMC Gastroenterol.

[23] Angelidis A.S, Tirodimos I, Bobos M, Kalamaki M.S, Papageorgiou D.K, Arvanitidou M (2011). Detection of *Helicobacter pylori* in raw bovine milk by fluorescence in situ hybridization (FISH). Int. J. Food Microbiol.

[24] Bianchini V, Recordati C, Borella L, Gualdi V, Scanziani E, Selvatico E, Luini M (2014). Helicobacteraceae in bulk tank milk of dairy herds from Northern Italy. BioMed. Res. Int.

[25] Mousavi S, Dehkordi S.F, Rahimi E (2014). Virulence factors and antibiotic resistance of *Helicobacter pylori* isolated from raw milk and unpasteurized dairy products in Iran. JVATiTD.

[26] Ghasemian Safaei H, Rahimi E, Zandi A, Rashidipourb A (2011). *Helicobacter pylori* as a zoonotic infection:the detection of *Helicobacter pylori* antigens in the milk and faeces of cows. J. Res. Med. Sci.

[27] Dunne C, Dolan B, Clyne M (2014). Factors that mediate colonization of the human stomach by *Helicobacter pylori*. World J. Gastroentero.

[28] Modolo L.V, de Souza A.X, Horta L.P, Araujo D.P, de Fatima A (2015). An overview on the potential of natural products as ureases inhibitors:A review. J. Adv. Res.

[29] Jiang X, Doyle M.P (1998). Effect of environmental and substrate factors on survival and growth of *Helicobacter pylori*. J. Food Protect.

[30] Kianpour F, Mehdipour S.Z, Mazroee M.A, Kazemeini H.R, Rahimi E, Jafari A (2014). Prevalence of *Helicobacter pylori* in bufflo milk in Iran. TLS.

[31] Turutoglu H, Mudul S (2002). Investigation of *Helicobacter pylori* in raw sheep milk samples. J. Vet. Med. B. Infect. Dis. Vet. Pub. Health.

[32] Oliver J.D (2005). The viable but nonculturable state in bacteria. J. Microbiol.

[33] Stevenson T.H, Castillo A, Lucia L.M, Acuff G.R (2000). Grow of *Helicobacter pylori* in various liquid and plating media. Lett. Appl. Microbiol.

